# Physical Properties of an Ag-Doped Bioactive Flowable Composite Resin

**DOI:** 10.3390/ma8084668

**Published:** 2015-07-24

**Authors:** Hiba Kattan, Xanthippi Chatzistavrou, James Boynton, Joseph Dennison, Peter Yaman, Petros Papagerakis

**Affiliations:** 1 Department of Orthodontics and Pediatric Dentistry, School of Dentistry, University of Michigan, Ann Arbor, MI 48109, USA; E-Mails: kattan@umich.edu (H.K.); xchatzis@umich.edu (X.C.); jboynton@umich.edu (J.B.); 2 Department of Cariology, Restorative Sciences, and Endodontics, School of Dentistry, University of Michigan, Ann Arbor, MI 48109, USA; E-Mails: dennison@umich.edu (J.D.); pyam@umich.edu (P.Y.)

**Keywords:** silver doped bioactive glass, flowable resin composite, depth of cure, biaxial flexural strength, polymerization shrinkage, *Streptococcus mutans*, *Lactobacillus casei*

## Abstract

The aim of this work was to study the physical and antibacterial properties of a flowable resin composite incorporating a sol-gel derived silver doped bioactive glass (Ag-BGCOMP). The depth of the cure was calculated by measuring the surface micro-hardness for the top and bottom surfaces. The volumetric polymerization shrinkage was measured by recording the linear shrinkage as change in length, while the biaxial flexural strength was studied measuring the load at failure. The antibacterial properties of the samples were tested against *Streptococcus mutans* (*S. mutans*) and *Lactobacillus casei* (*L. casei*). The measured values were slightly decreased for all tested physical properties compared to those of control group (flowable resin composite without Ag-BG), however enhanced bacteria inhibition was observed for Ag-BGCOMP. Ag-BGCOMP could find an application in low stress-bearing areas as well as in small cavity preparations to decrease secondary caries. This work provides a good foundation for future studies on evaluating the effects of Ag-BG addition into packable composites for applications in larger cavity preparations where enhanced mechanical properties are needed.

## 1. Introduction

Composite resins are recently considered the most commonly used tooth-colored intra-coronal restoration [1]. Flowable composites have similar composition to that of traditional composite with less of the filler content, which subsequently reduces their viscosity [2]. Flowable composite has a wide variety of applications; it can be used as a pit and fissure sealant, preventive resin restoration (PRR), cavity liner, restoration repair, small and a pediatric restoration [3]. The use of a flowable composite as a liner has an advantage of reducing the amount of microleakage [4]. Flowable composite is also an acceptable treatment to be used as a preventive resin restoration [5]. Although composite fillings have long-term clinical success, failures most commonly result from secondary caries, which can lead to weakening of enamel around the restoration, marginal breakdown and subsequently loss of the restoration [6,7,8]. In fact, 50%–70% of a dentist’s work is to replace failed restorations [9]. Several attempts have been made to overcome this problem by adding antimicrobial materials, such as fluoride and chlorhexidine to the resin matrix in order to prevent and hopefully eliminate recurrent decay and provide antimicrobial properties. These materials have not been widely used because of diminishing fluoride release and uptake with time, as well as their short-term effect [10,11]. Quaternary ammonium and metal particles were also used as additives. The incorporation of these additives has been proven to affect the bond strength and the physical properties of the modified composites [12,13]. Silver and zinc-oxide were also some of the successfully incorporated additives. In 2014, Kasraei tested the effect of adding 1% silver and 1% zinc-oxide nanoparticles into flowable composite, and resulted in a decrease in *Streptococcus mutans* and *Lactobacillus* reducing recurrent caries [14].

The idea of developing bioactive resin composite able to elicit a bioactive surface around the micro-gaps of the restoration and complete sealing between composite and dentinal wall could advance the currently used clinical approaches. This type of sealing could prolong the life of restorations by eliminating secondary caries and micropenetration of oral bacteria [15]. It has been observed that when composite is inserted into a cavity and bonded into more than one dentinal wall, the tooth-bond interface goes into contraction stresses that can exceed the bond strength and may lead to a separation. Thus, a path for microorganisms is created and, subsequently, failure of the restoration may occur if it takes place at an exposed margin [16,17]. The development of composites with bioactive behavior being capable of forming an apatite layer on their surface could provide the required conditions, which may improve its bond with the dentinal wall [18]. Moreover, filler content in composites is one of the factors affecting the amount of polymerization shrinkage [19]. Decrease of filler weight in flowable composites compared to those in conventional composite result in more polymerization shrinkage [19]. Therefore, the fabrication of new resin composite material incorporating bioactive and anti-bacterial particles could prevent secondary caries but also could retain key physical properties to allow satisfactory polymerization shrinkage, depth of cure, and bond strength.

The successful incorporation of Ag as ion within the glass allows simultaneously a dual antibacterial and remineralizing activity with a controlled releasing process. These properties could be of important impact for a dental resin composite as they could lead to the remineralization of the microgaps under a bacterial free environment in the treated area. Recently, a new one-step self-adhering flowable resin composite (modified-composite) was developed incorporating an Ag-doped bioactive glass (Ag-BG). Ag-BG was previously evaluated for its effective antimicrobial activity, the potential to form an apatite phase and the total bond strength [18,20,21]. The aim of this study was to characterize key mechanical, physical and bactericidal properties of the newly developed Ag-BG flowable composite (Ag-BGCOMP). It was observed that these new Ag-BG composites (Ag-BGCOMPs) present bactericidal and bioactive properties while retaining their good mechanical characteristics.

## 2. Materials and Methods

### 2.1. Fabrication

Our previous work describes in detail the fabrication of Ag-BG [21]. Briefly, the fabrication Ag-BG is based on incorporating the sol-gel bioactive glass 58S (SiO_2_ 58-CaO 33-P_2_O_5_ at 9 wt %) into the solution stage of the sol-gel glass in the system SiO_2_ 60-CaO 6-P_2_O_5_ 3-Al_2_O_3_ 14-Na_2_O 5-K_2_O 5-Ag_2_O at 7 wt %. The resulting composite solution follows an aging process at 60 °C, then drying at 180 °C and finally stabilization at 700 °C. The final sol-gel derived Ag-doped bioactive glass (Ag-BG) is in the system as SiO_2_ 58.6-CaO 24.9-P_2_O_5_ 7.2-Al_2_O_3_ 4.2-Na_2_O 1.5-K_2_O 1.5-Ag_2_O 2.1 at wt % [21]. The fabricated Ag-BG glass is in powder form with particle size around ~25 μm.

Specimens with 10 wt % of Ag-BG within the flowable composite (Ivoclar Vivadent, Tetric EvoFlow^®^ Filling Material A1, Amherst, NY, USA) were formulated prior to the initiation of the test methods. The concentration of 10 wt % Ag-BG was chosen based on the results of our previous work with 5 and 15 wt % Ag-BG [18]. The specific concentration is expected to present optimum bactericidal and mechanical properties. Ag-BG in powder form was incorporated manually until it appeared homogenous for approximately 5min using a stainless steel spatula. Particles weren’t silanized. Samples without Ag-BG incorporated were used as controls (controls: Ag-BG at 0 wt %).

**Figure 1:**
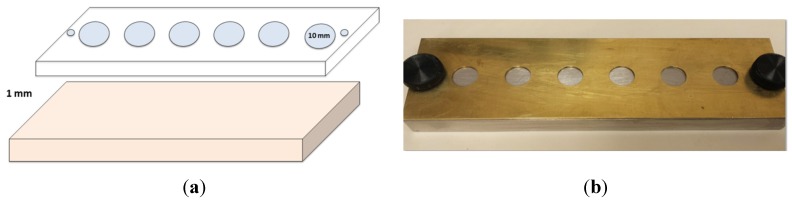
(**a**) Layout of the two parts of brass mold; (**b**) Brass mold assembly.

A split brass mold was fabricated, with each cylinder 10 mm in diameter and 1 mm deep (Figure 1a,b). Molds were lubricated using a releasing agent (Al-Cote, Dentsply International, York, PA, USA) to facilitate separation of samples and prevent composite adhesion to the metal base. In order to prevent the formation of the resin rich layer after curing, a clear plastic strip was placed over the metal base. Mold was assembled and secured using metal screws (Figure 1b). Composite was then injected into each one of the holes in the brass mold and a wax spatula was used to remove excess composite. Another clear strip was placed on top of the samples, followed by a 1 mm thick glass slide to allow standardization of sample thickness and distance from the curing tip. Each sample (*n* = 12) was cured for 20 s according to manufacturer’s recommendation (using a halogen curing light (Valo, Ultradent, South Jordan, UT, USA)) operating with the wavelength of 400–500 nm and the intensity of about 1000 mW/cm^2^. The light cure unit was re-tested for intensity after every five samples using a radiometer (Cure Rite^®^, DENTSPLY International Inc., Milford, DE, USA). Molds were cleaned after each use with isopropyl alcohol gauze (70%) to remove any remaining residues.

After curing, each side of the samples were polished for 1 min in a rotary polishing machine (MAGER Scientific, Dexter, MI, USA) with 600-grit wet silicon carbide paper (MAGER Scientific, Dexter, MI, USA) and a rotation speed of 102 rpm. Additional polishing was done using 800 grit silicon carbide paper for 3 min to achieve a glossy surface. Samples were rinsed with tap water and kept in a small dark container for 24 h at room temperature prior to testing.

### 2.2. Depth of Cure

Microhardness testing was performed using a Tukon 2100B-testing machine (Wilson Instrument, Northwood, MA, USA) after 24-hour of dry storage. Each sample (*n* = 12) was positioned and stabilized. Vicker diamond indenting tool and a load of 200 g was applied to the sample’s surface with a dwell time of 15 s. All measurements were made within a 4 mm radius of the specimens’ centers. Three hardness measurements were recorded and averaged for each surface of the specimens. Top/bottom ratio of each sample was calculated and 80% was set as the acceptable depth of cure [22].

### 2.3. Polymerization Shrinkage

Linear polymerization shrinkage was measured using a linometer (Kaman industrial Corp, Colorado Springs, CO, USA). A three-point calibration was done on the hardware to the minimum, midpoint, and maximum distance to be measured (0 µm, 500 µm, 1000 µm). Finally, a 21-point calibration was done on the software, correlating change in probe response every 50 µm, from the minimum distance of 0 µm through maximum distance of 1000 µm. Samples (*n* = 12) were prepared and tested in a dark room, at room temperature to prevent any additional polymerization. The Linear displacement was calculated as change in length (∆L), and the percentage of linear shrinkage (Lin%) was calculated by using the following equation:

Lin% = (∆*L*/*L* + ∆*L*) × 100 (*L* is the sample’s thickness after polymerization)

Lin% was then converted to a volumetric percentage (Vol %) using the following equation:

Vol % = 3 Lin% − 0.03 (Lin%)^2^ + 0.0001 (Lin%)^3^

### 2.4. Biaxial Flexural Strength

A cylindrical stainless steel ring with a diameter of 9 mm and thickness of 1 mm was used to fabricate composite samples for biaxial flexural strength testing. A total of twelve samples were made for each group (*n* = 12). Samples were then stored in a dark container at room temperature for 24 h. Biaxial flexural strength was measured using a universal testing system (Instron 4502, Instron Corp., Canton, MA, USA). A speed of 0.50 mm/min was used and the maximum load (*P* in Newtons) applied on the specimen prior to fracture was measured. The biaxial flexural strength (*S* in MPa) was calculated using the following equation:

*S* = −0.2387*P*(*X* − *Y*)*d*^2^

where *X* and *Y* are constant parameters that are calculated from the experimental settings.

### 2.5. Antibacterial Properties

The extracts of the samples were collected after immersion of both control and Ag-BGCOMP in PBS for up to 22 days. The pH was monitored as well. The bactericidal properties of the extracts were tested against *S. mutans* ATCC 25175 and *L. casei* ATCC 15008 which are basic cariogenic bacteria. A single colony of bacteria was inoculated in nutrient broth and grown overnight at 37 °C. After adjusting to an optical density equivalent to 10^8^ cells per ml in PBS, sequential tenfold dilutions were added to tubes containing equal volumes of the extracts. The effect of the materials’ extracts on bacterial growth was assayed by colony forming units (CFU) on nutrient agar plates after 24 h growth.

### 2.6. Statistical Analysis

The results were analyzed by using Statistical Package for the Social Sciences (SPSS). The statistically significant difference between control and Ag-BGCOMP was determine using t-test with the level of significance at *p* = 0.05.

## 3. Results

**Figure 2:**
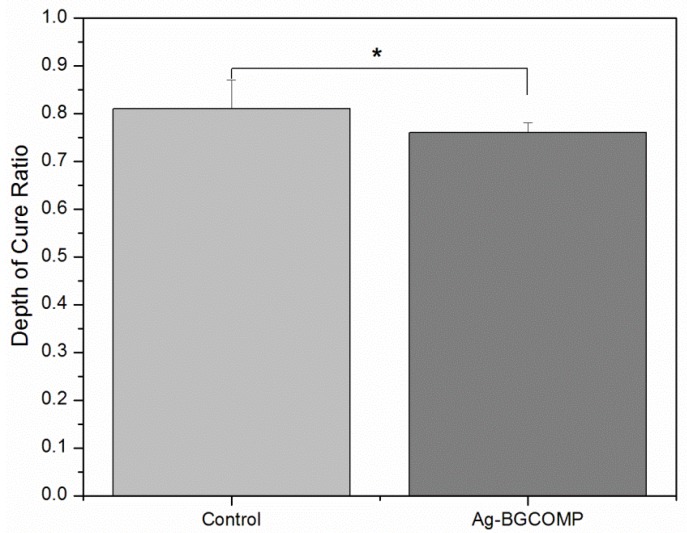
Depth of cure ratio mean value for control and Ag-BGCOMP. * = statistically significant difference (*p* = 0.02) between the two groups. Error bars represent the standard deviation (SD) calculated in each group.

Figure 2 shows the mean and standard deviation of depth of cure ratio for control and Ag-BGCOMP groups. There was a significant difference at the measured values between the two groups (*p* = 0.02). The control group had depth of cure ratio at 0.81 ± 0.06 just above the 0.8 acceptable ratio value, while the Ag-BGCOMP group had slightly less at 0.76 ± 0.02.

**Figure 3:**
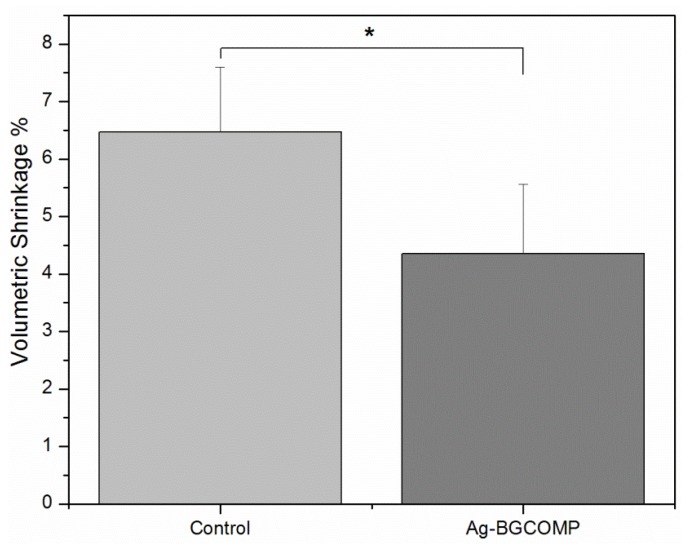
Volumetric polymerization shrinkage % mean value for control and Ag-BGCOMP. * = statistically significant difference (*p* = 0.0002) between the two groups. Error bars represent the standard deviation (SD) calculated in each group.

Figure 3 shows the mean values of the volumetric % shrinkage due to the polymerization process both for control and Ag-BGCOMP. There was a significant difference between the two groups (*p* = 0.0002). The incorporation of Ag-BG powder at 10 wt % decreases the volumetric polymerization shrinkage of the resin composite by around ~30% (from 6.47% shrinkage to 4.35%).

**Figure 4:**
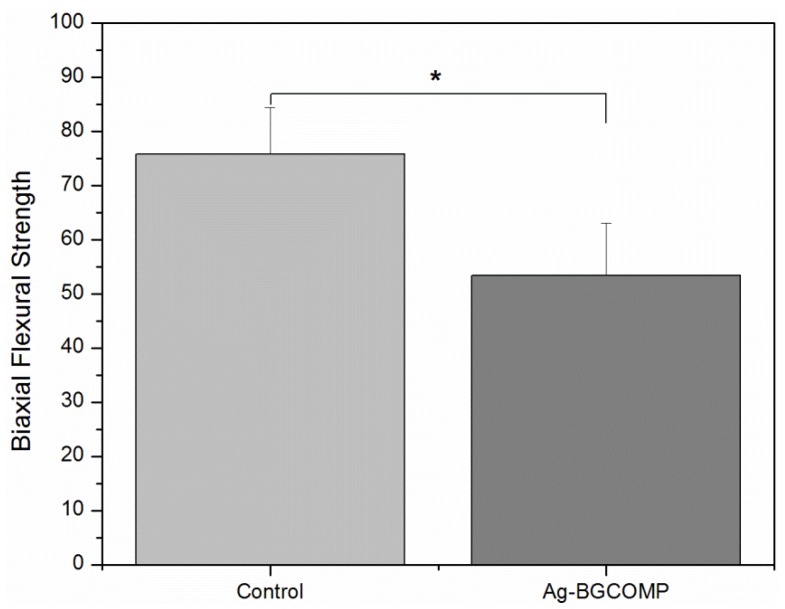
Biaxial flexural strength of control and Ag-BGCOMP. * = statistically significant difference (*p* = 0.0001) between the two groups. Error bars represent the standard deviation (SD) calculated in each group.

Figure 4 shows the mean values of biaxial flexural strength for control and Ag-BGCOMP groups. There is a significant difference between the measured values of the two groups (*p* = 0.0001). A decrease of 30% in the biaxial strength is observed for Ag-BGCOMP compared to control.

All fractures were initiated in the center of each sample. Fracture patterns of different groups were different. All control samples generated two pieces while the Ag-BGCOMP samples generated three equal pieces. Table 1 shows a summary of all measured values with the respective standard deviations.

**Figure 5:**
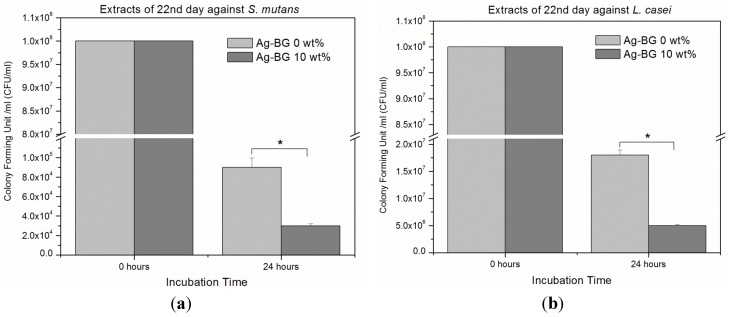
Antibacterial activity of the extracts of control and Ag-BGCOMP samples, after 22 days of immersion in PBS, shows significant bacteria growth inhibition against (**a**) *S. mutans* and (**b**) *L. casei* (error bars = ±SD; *n* = 3, * *p* < 0.05).

Figure 5a,b present the capability of samples’ extracts after 22 days of immersion in Phosphate Buffered Saline (PBS), to inhibit bacteria growth. Bacteria growth inhibition against *S. mutans* and *L. casei* is significantly higher for the extracts of Ag-BGCOMP compared to control (*p* < 0.05). The pH value of the extracts after 22 days was observed at neutral level of 7.4.

**Table 1:** Mean values (SD) of the three properties of control and a silver doped bioactive glass (Ag-BGCOMP).

Parameter	Control	Ag-BGCOMP	*p* Value
Depth of cure ratio	0.81 (0.06)	0.76 (0.02)	S*
Volumetric Polymerization shrinkage (SD) [%]	6.47 (1.13)	4.35 (1.21)	S*
Biaxial flexural strength (SD) [MPa]	75.82 (8.59)	53.37 (9.43)	S*

S* = Significant Difference (*p* < 0.05).

## 4. Discussion

The aim of this study was to evaluate the mechanical, physical and bactericidal characteristics of a Ag-BG flowable composite (Ag-BGCOMP)—in particular, the depth of cure, polymerization shrinkage and biaxial flexural strength of a modified flowable composite that contains 10% wt. Ag-BGs were determined as well as the antibacterial properties against *S. mutans* and *L. casei*.

The biocompatibility of composite can be related directly to its ability to polymerize. The uncured part of composite restorations can easily dissolve in the oral cavity and subsequently lead to secondary caries. In addition, leaching unreacted monomers from the uncured material can cause local tissue irritations [23,24].

Our results showed a significant difference in depth of cure ratio between the two groups. Ag-BGCOMP showed only 5% less depth of cure than the control, which is not clinically significant if the control group cured for more than 80%. Moreover the non-clinically significant difference was also anticipated based on our previous work on the bond strength values for 5 and 15 wt % Ag-BG [18]. Non-significant differences to the control were found for these values as well, which is expected to also be the case for the 10 wt % of Ag-BG. The low depth of cure of both materials tested may be related to factors such as its chemical composition, light cure intensity and curing time [22,25,26]. Materials with higher opacity can lower the depth of cure ratio, as the light energy cannot penetrate the sample enough to fully promote polymerization reaction [27]. This might explain the difference in depth of cure between the two groups as the incorporation of Ag-BG particles may have increased the opacity of the composite and subsequently decreased the depth of cure. Because of this drop the use of Ag-BGCOMP could be proposed as a liner or as a restorative material in shallow cavities. Thus, the possibility of leaching the monomer in the oral cavity and leading to local tissue irritation may be decreased.

Polymerization shrinkage measures the dimensional change of the material during polymerization. It has been observed that polymerization shrinkage and shrinkage stress increased over time. Shrinkage depended on filler load, type of filler particles, monomer system, and the existence of pre-polymerized particles. There was a strong linear correlation between polymerization shrinkage and shrinkage stress [28]. Our results showed higher shrinkage of the control compared to the Ag-BGCOMP samples. This can be attributed to the incorporated Ag-BG particles and the increase of the filler load, leading to decrease of the flow and subsequently decrease of the polymerization shrinkage [28,29]. The lower shrinkage of Ag-BGCOMP could be also attributed to the unsilanized incorporated particles. However, the Ag-BG containing composites are designed to have ions leaching out of the incorporated particles, because of that it is recommended the use of unsilanized particles [30]. In an opposite case a decrease on the antimicrobial and remineralizing activity of the silanized particles would be expected.

Composite resin restorations are clinically subjected to stress due to forces from mastication [3]. When a material is subjected to increased masticatory stresses, it is critical that this material has high flexural strength [31]. In the present study, biaxial flexural strength was used instead of the three- point test to eliminate the additional light exposure necessary to cure the composite rods for the three -point test when fabricating the samples. Our results showed that Ag-BGCOMP shows less flexural strength than the control group. This may occur due to the incorporated air voids while hand mixing the flowable composite with Ag-BG glass. Thus, the fabrication process itself could weaken the composite and it may result in a more brittle material. This drawback could be overcome simply by applying a more thorough commercial mixing procedure to remove the trapped air. Nevertheless, Ag-BGCOMP may have to be applied selectively and not be placed on load-bearing areas. Moreover, it could be used as a cavity liner instead of a final restoration in high stress areas. Another alternative idea to increase the strength of the composite could be the incorporation of nano-size Ag-BG and/or coupling the Ag-BG particles during the fabrication of the resin composite [32].

The addition of Ag-BG leads to significantly enhanced antibacterial properties against both *S. mutans* and *L. casei* compared to the control, while the pH remains neutral even after 22 days of soaking period. This result confirms that the observed bactericidal activity is not due to changes in the pH value. On the other hand, release of silver ions is expected to occur from the Ag-BGCOMP samples and last at least up to a month, as is the case for Ag-BG particles [20]. This behavior is explained by the fact that silver ions show antibacterial action following several mechanisms, including interacting with thiol groups in proteins and interfering with DNA replication [33].

## 5. Conclusions

Our study presents the fabrication of an antibacterial and bioactive resin composite incorporating an Ag-doped bioactive glass into a flowable composite. The depth of cure ratio was found to be slightly lower for the Ag-BGCOMP compared to control; however, the difference is not considered clinically significant. Improved polymerization shrinkage was observed for the Ag-BGCOMP; however, a decrease of 30% occurred on the flexural strength. Finally, the antibacterial properties of the new Ag-BGCOMP resin composite were found to be significantly higher than the control. The results of this work contributes to the development of new resin composites that can inhibit the caries decay process and increase the lifetime of dental restorations.
